# Wealth and Disability in Later Life: The English Longitudinal Study of Ageing (ELSA)

**DOI:** 10.1371/journal.pone.0166825

**Published:** 2016-11-22

**Authors:** Juliana Lustosa Torres, Maria Fernanda Lima-Costa, Michael Marmot, Cesar de Oliveira

**Affiliations:** 1 Programa de Pós-graduação em Saúde Pública, Universidade Federal de Minas Gerais, Belo Horizonte, Minas Gerais, Brazil; 2 Rene Rachou Research Center, the Oswaldo Cruz Foundation in the State of Minas Gerais, Belo Horizonte, Minas Gerais, Brazil; 3 Research Department of Epidemiology and Public Health, University College London, London, United Kingdom; Maastricht University, NETHERLANDS

## Abstract

We examined wealth inequalities in disability, taking into account the effect of both depression and social support among older English adults using data from 5,506 community-dwelling people aged 50 years and over from the English Longitudinal Study of Ageing (ELSA). Disability was measured as self-reported limitations in the Basic Activities of Daily Living (ADL) and Instrumental Activities of Daily Living (IADL). Depressive symptomatology was measured using the 8-item Center for Epidemiological Studies-Depression (CES-D) scale. Social support was assessed by marital status and frequency of contact with friends, relatives or children. Multinomial logistic regression models were used to assess the role of social support and depressive symptoms on disability by total household wealth, which is a measure of accumulated assets over the course of life. Our findings showed that the poorest men with disability were more likely to live without a partner and have no weekly contact with children, family or friends compared to the wealthiest. Among women with disability, the poorest were more likely to report loneliness and have no partner while the wealthiest and the intermediate groups were more likely to be living with a partner. There was a strong inverse dose-response association between wealth and depressive symptoms among all participants with disability. This study shows a clear wealth gradient in disability among older English adults, especially for those with elevated depressive symptoms.

## Introduction

Socioeconomic inequality in health is a key public health concern [1] with many studies showing gradients in physical ill health and mortality by socioeconomic position [2]. The gradient in the association between socioeconomic status (SES) and functioning is well documented, with individuals from higher SES experiencing better functioning [3]. The association between SES, negative emotions and depressive symptoms has also been investigated [4, 5]. In most conceptual models, possible pathways connecting low SES with poor health can be distilled roughly into two categories: stress and concomitant psychological distress, and psychological and social resources [6].

The literature also reports that some psychosocial factors i.e. measurements that potentially relate psychological phenomena to the social environment and to pathophysiological changes [7] are associated with disability. These psychosocial factors included depression [8,9], loneliness [10], social networks [11] and social support [12]. Measures of social networks have been shown to be associated with SES, in the sense that individuals in higher socioeconomic groups are more likely to be married, have more friends and report higher levels of social support (more emotional support and less negative aspects of close relationships) [13].

Depression is a major contributor to disability, accounting for 4.4% of total disability-adjusted life years (DALY) globally [14] and it has consistently been shown to be a strong predictor of physical limitation and difficulty performing activities of daily living (ADLs) in community-dwelling adults [15]. Its relationship with SES has been explored previously [5].

Previous research has shown an inverse SES gradient in depressive symptoms and poor physical functioning [5]. However, it is still unclear from the literature in health inequalities whether the association between psychosocial factors and functioning is consistent throughout different SES groups or whether there are interactions. This is because poor social resources are hypothesized to lead to disability by influencing health-damaging behaviours and psychological and physiological systems [13], and, on the other hand, high SES groups have the potential to attenuate these effects due to less stress levels accumulated in the life course [6]. In addition, most of the literature considers linearity in the associations among SES, psychosocial pathways, and health (mainly functioning), reporting adjusted coefficients that might represent merely the average coefficients across the SES categories. That is, psychosocial factors could potentially have a greater impact at certain levels of SES than at others, or different psychosocial factors may be important for determining health of lower versus higher SES individuals [16]. For example, men in the lowest SES group are less likely to live with a partner [13] and, consequently, experiencing more stress since their social support is heavily focused on their spouses who are less present. Therefore, the main aim of this study is to explore socioeconomic inequalities in disability, taking into account the effect of both depression and social support among different socioeconomic groups of older English adults.

## Methods

### Study Population

The English Longitudinal Study of Ageing (ELSA) is a representative sample of the population aged 50 and over, living in private households in England. Its participants were recruited from households that had earlier participated in the Health Survey for England. ELSA is a wide data source including information on sociodemographic and health characteristics, social participation and biomarkers and a detailed description of the study can be found elsewhere [17]. Of 9,169 ELSA core participants who took part in wave 6 (2012–13), 5,506 had complete data on all study variables. Those who were excluded tended to be older females with disability from the lowest socioeconomic group (p<0.001). Wave 6 was used for this analysis because we wanted to reflect the current ELSA participants’ wealth and disability circumstances.

### Assessment of disability

Disability was measured as self-reported limitations in the Basic Activities of Daily Living (ADL) and Instrumental Activities of Daily Living (IADL) [18]. ADL included six activities: dressing, walking across a room, bathing or showering, eating, getting in or out of bed, using the toilet. IADL included seven activities: using a map to get around in a strange place, preparing a hot meal, shopping for groceries, making telephone calls, taking medications, doing work around the house or garden and managing money. Disability was defined as having limitation in one or more activities, including ADL and IADL.

### Assessment of household wealth

Total non-pension household wealth included financial wealth (savings and investments), the value of any home and other property (less mortgage), the value of any business assets and physical wealth such as artwork and jewellery owned by the household (i.e., a single respondent or a responding couple along with any dependent individuals) minus any debt. Wealth is the most robust indicator of socioeconomic circumstances in ELSA, and has been found to be more strongly associated with the risk of death than any other socioeconomic position indicator at older ages [19]. The estimation of this variable was based on 22 different wealth and debt components, which were either observed or imputed. A detailed description of wealth and its components can be found at: http://bit.ly/1yrRgHd and http://bit.ly/1awp6iZ.

### Assessment of social support

Marital status was categorized in having a partner and not having a partner (single, widowed, separated or divorced). The frequency of contact at a weekly rate or more often (either face to face, over the phone, email or text messages) with friends, relatives or children who did not live with the respondent was assessed and used as a dichotomous variable (yes/no). The objective was to identify respondents who had no frequent contact with anyone outside their household. Positive social support received by children/friends/family was measured by three questions on participant’s perceptions of support availability and used as a dichotomous variable (high/low). By each network type, we defined that the participants had positive social support if they reported ‘a lot’ in three questions or ‘a lot’ in two questions and ‘some’ in one. Then, we combined the three network types. High positive social support was defined by having support in at least one network type and low positive social support by having no support of each network type.

### Assessment of loneliness

Loneliness was assessed by the Three-Item Loneliness Scale [20] derived from the 20-item revised UCLA loneliness scale [21], with reliability reported as 0.72 [20]. The scale includes questions about feeling lack of companionship, feeling left out and feeling isolated from others. The three-point response scale ranged from 1 (hardly ever/never) to 3 (often) and a score ranging from 1 to 9 was obtained and divided into tertiles: those in the highest loneliness tertile were compared to the intermediate/lower tertile.

### Assessment of depressive symptoms

Depressive symptoms were measured by the shortened version of the Center for Epidemiological Studies-Depression (CES-D) scale [22]. The scale included eight questions about depressive symptoms experienced during the week before the ELSA interview. A dichotomous variable distinguishing between those with and without depressive symptoms was derived, considering the validated cut-off point of four or more depressive symptoms [23].

### Covariates

Potential confounders included in this analysis were age and number of comorbidities. The number of comorbidities was assessed by self-reported doctor diagnosed chronic diseases, including diabetes, cancer, stroke, arthritis, lung disease, Parkinson and cardiovascular diseases (high blood pressure, angina, heart attack, heart failure, heart murmur or heart rhythm). The number of comorbidities was categorized into none, one or two or more.

### Statistical analysis

Univariate analysis was conducted first, using Pearson’s chi-square test for categorical variables and ANOVA for continuous variables. We used Multinomial Logistic Regression to estimate the odds ratio (OR) and their 95% confidence intervals to assess the association of psychosocial aspects with disability and wealth. Four outcomes were considered in the multinomial logistic regressions: without disability (reference category), disability in highest wealth tertile (Wealthiest), disability in intermediate wealth tertile (Intermediate) and disability in lowest wealth tertile (Poorest). This type of analysis was used to firstly test the effect of disability (with/without) and secondly the interaction between disability and household wealth. Multivariate analysis was performed using sequential models. First, we estimated the association between psychosocial aspects and disability and wealth by adjusting for age. Then we added the number of comorbidities and lastly, depressive symptoms. The analysis was stratified by gender, as psychosocial factors are different between men and women, using STATA 13.0 (Stata Corp LLP, College Station, TX).

### Ethics Approval and Informed Consent

All participants gave written informed consent. The English Longitudinal Study of Ageing has been approved by the National Research Ethics Service (London Multicentre Research Ethics Committee (MREC/01/2/91)).

## Results

Of 9,169 ELSA core participants who took part in wave 6 (2012–13), we had information about wealth and disability outcomes for 8,945, and among those, complete information regarding psychosocial variables for 5,506, which were included in the current analysis. Characteristics of the study population by ADL/IADL limitation are showed in Table 1. The prevalence of disability was 20.9% and among these, 47.6% were in the lowest socioeconomic group. The mean age of participants was 66.0 years (SD = 8.4), with a majority of these female (54.1%) and 37.6% with one comorbidity. Depressive symptoms were reported in 10.2%. The group with disability was poorer (47.6%), with a majority of females (57.4%), with two or more comorbidities (50.9%), more depressive symptoms (24.3%), were not living with a partner (35.5%) and reported more loneliness (39.5%).

**Table 1:** Characteristics according to disability status at wave 6 (2012–13) of 5,506 participants aged 50 and older from the English Longitudinal Study of Ageing (ELSA).

Characteristic	All (n = 5,506)	Disability		*P* value
		Yes (n = 1,153)	No (n = 4,353)	
Age (years), mean (SD)	66.0 (8.4)	68.9 (9.0)	65.3 (8.1)	<0.001
Women, (%)	54.1	57.4	53.3	0.012
Household wealth, (%)				<0.001
Wealthiest	33.4	21.9	36.5	
Intermediate	33.6	30.4	34.4	
Poorest	33.0	47.6	29.1	
Number of comorbidities*, (%)				<0.001
None	36.5	14.7	42.3	
One	37.6	34.4	38.5	
Two or more	25.8	50.9	19.2	
Marital status (not living with partner), (%)	32.7	35.5	32.0	0.025
No weekly contact with friends, family or children, (%)	5.2	5.5	5.2	0.689
Loneliness (highest tertile), (%)	28.2	39.5	25.2	<0.001
Low social support from friends, family or children, (%)	31.3	33.0	30.9	0.154
Depressive symptoms (≥4 CES-D symptoms), (%)	10.2	24.3	6.5	<0.001

* Self-reported doctor diagnosed chronic diseases = diabetes, cancer, stroke, arthritis, lung disease, Parkinson and CVD conditions (high blood pressure, angina, heart attack, heart failure, heart murmur or heart rhythm)

Table 2 shows the prevalence of each covariate by disability status and household wealth tertile among men and women. Among men, the poorest reported more elevated depressive symptoms (28%), were not currently living with a partner (43.1%), were more likely to report no weekly contact with their children, family or friends (9.3%) and experienced more loneliness (36.4%). Among women, the same pattern was observed for the poorest: more depressive symptoms (38%), not currently living with a partner (52.5%) and experienced more loneliness (49.7%). Additionally, we tested whether there was any difference in disability severity (number of activities reported with limitation) across wealth groups and we found a statistically significant gradient among women. The prevalence of limitations in four or more activities reported by the wealthiest, intermediate and the poorest women were 10.2%, 22% and 29.6% respectively (data not shown).

**Table 2:** Characteristics according to disability status by household wealth in 5,506 men and women, the English Longitudinal Study of Ageing (ELSA), wave 6 (2012–13).

Characteristic	Without disability % (n = 4,353)	Disability			*P* value
		Wealthiest % (n = 253)	Intermediate % (n = 351)	Poorest % (n = 549)	
***Men***					
Age, mean (SD)	66.0 (8.3)	71.2 (9.0)	71.4 (8.9)	68.4 (9.1)	<0.001
Number of comorbidities*					<0.001
None	39.2	19.8	13.8	11.1	
One	40.1	39.6	33.1	32.0	
Two or more	20.7	40.6	53.1	56.9	
Marital status (not living with partner)	28.1	26.4	20.6	43.1	<0.001
No weekly contact with friends, family or children	5.0	0.9	5.0	9.3	0.008
Loneliness (highest tertile)	22.5	34.0	31.9	36.4	<0.001
Low social support from friends, family or children	39.6	44.3	38.1	39.6	0.769
Depressive symptoms (≥4 CES-D symptoms)	4.2	11.3	15.0	28.0	<0.001
***Women***					
Age, mean (SD)	64.5 (7.8)	69.4 (8.7)	68.9 (8.4)	66.9 (8.9)	<0.001
Number of comorbidities*					<0.001
None	45.0	15.7	18.9	13.3	
One	37.1	41.5	36.1	30.6	
Two or more	17.9	42.9	45.0	56.2	
Marital status (not living with partner)	35.4	21.1	26.2	52.5	<0.001
No weekly contact with friends, family or children	5.4	4.1	5.2	5.3	0.931
Loneliness (highest tertile)	27.6	36.7	37.2	49.7	<0.001
Low social support from friends, family or children	23.2	29.9	24.6	28.7	0.058
Depressive symptoms (≥4 CES-D symptoms)	8.5	15.0	18.9	38.0	<0.001

* Self-reported doctor diagnosed chronic diseases = diabetes, cancer, stroke, arthritis, lung disease, Parkinson and CVD conditions (high blood pressure, angina, heart attack, heart failure, heart murmur or heart rhythm)

**Fig 1:**
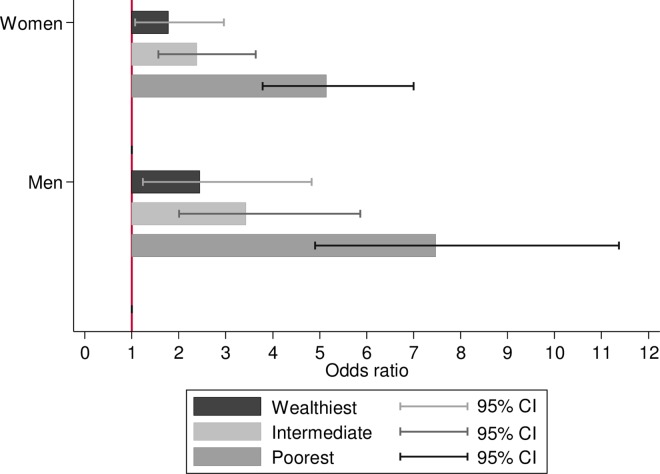
Fully adjusted odds ratios and 95% CI of depressive symptoms among men and women with disability, according to wealth tertiles. The English Longitudinal Study of Ageing, wave 6 (2012–13).

The results from the multinomial logistic regression showed that the poorest men with disability were more likely to have no partner (OR = 1.78; 95% CI 1.29, 2.45) and to report no weekly contact with their children, family or friends (OR = 1.79; 95% CI 1.01, 3.16). The wealthiest men experienced more loneliness (OR = 1.59; 95% CI 1.01, 2.49), even after adjusting for multiple variables. Among women, the poorest with disability were more likely to report loneliness (OR = 1.52; 95% CI 1.15, 2.01) and not having a partner (OR = 1.88; 95% CI 1.44, 2.44). On the other hand, the wealthiest and the intermediate groups were more likely to be living with a partner. There was a strong inverse dose-response association between wealth and elevated depressive symptoms among men and women with disability (Table 3). The odds ratios adjusted for age, chronic diseases and psychosocial characteristics and their 95% CIs for this association are displayed in Fig 1. Participants without disability are the reference category.

**Table 3:** Multinomial Logistic regression analyses of older adults without disability (n = 1,153) aged 50 years and over in England by wealth tertiles, the English Longitudinal Study of Ageing (ELSA), wave 6 (2012–13).

	Model^a^			Model^b^		
	Disability			Disability		
	Wealthiest (n = 253) OR (95%CI)	Intermediate (n = 351) OR (95%CI)	Poorest (n = 549) OR (95%CI)	Wealthiest (n = 253) OR (95%CI)	Intermediate (n = 351) OR (95%CI)	Poorest (n = 549) OR (95%CI)
***Men***						
Marital status (not living with partner)	1.01(0.64–1.60)	**0.65 (0.43–0.99)**	**1.71 (1.26–2.33)**	1.02 (0.65–1.62)	0.66 (0.43–1.01)	**1.78 (1.29–2.45)**
No weekly contact with friends, relatives or children	0.25 (0.33–1.82)	1.50 (0.69–3.29)	**1.80 (1.05–3.10)**	0.25 (0.03–1.83)	1.52 (0.69–3.34)	**1.79 (1.01–3.16)**
Loneliness (highest tertile)	**1.80 (1.17–2.78)**	**1.75 (1.21–2.53)**	**1.72 (1.26–2.35)**	**1.59 (1.01–2.49)**	1.42 (0.97–2.11)	1.09 (0.77–1.55)
Low social support from friends, family or children	1.36 (0.90–2.04)	1.09 (0.77–1.55)	1.15 (0.85–1.56)	1.33 (0.88–2.01)	1.07 (0.75–1.53)	1.12 (0.82–1.53)
Depressive symptoms (≥4 CES-D symptoms)	-	-	-	**2.44 (1.24–4.83)**	**3.43 (2.00–5.86)**	**7.47 (4.90–11.37)**
***Women***						
Marital status (not living with partner)	**0.52 (0.34–0.79)**	**0.67 (0.48–0.95)**	**1.97 (1.53–2.54)**	**0.52 (0.34–0.79)**	**0.66 (0.47–0.94)**	**1.88 (1.44–2.44)**
No weekly contact with friends, family or children	0.94 (0.40–2.21)	1.11 (0.56–2.19)	0.84 (0.48–1.47)	0.94 (0.40–2.22)	1.13 (0.57–2.23)	0.90 (0.51–1.60)
Loneliness (highest tertile)	**1.59 (1.10–2.29)**	**1.65 (1.19–2.28)**	**2.22 (1.72–2.87)**	1.45 (0.99–2.11)	**1.42 (1.01–1.98)**	**1.52 (1.15–2.01)**
Low social support from friends, family or children	1.38 (0.94–2.02)	1.03 (0.73–1.49)	1.16 (0.88–1.54)	1.36 (0.93–2.00)	1.02 (0.71–1.45)	1.09 (0.82–1.47)
Depressive symptoms (≥4 CES-D symptoms)	-	-	-	**1.78 (1.07–2.96)**	**2.39 (1.57–3.64)**	**5.15 (3.78–7.00)**

Models using participants without disability as reference category: Model^a^ = adjusted for age, number of chronic diseases and psychosocial characteristics; Model^b^ = Model^a^ + depressive symptoms.

Bold: p<0.05

## Discussion

Our main findings showed a clear wealth gradient in disability in later life, with better levels of social resources among those who were better off. Depressive symptoms emerged as the most significant psychosocial indicator investigated. The poorest participants with disability reported more depressive symptoms and and this aspect was particularly severe among the poorest women. Loneliness was associated with functioning independently of wealth, but, after adjusting for depressive symptoms, this association lost strength and remained statistically significant only for some wealth groups. Finally, for both men and women the poorest were more likely to be without a partner, whilst the poorest men were more likely to report no weekly contact with friends, family or children, and poor women to be lonelier.

Current evidence on socioeconomic trends in the disability-free life expectancy of older ages in England supports the clear wealth gradient in disability found in this study. Older English adults in the least affluent areas spent more years with disability compared to those living in wealthier areas [24]. In the past three decades, income inequality in England increased steeply and it has been sustained at historically high levels [24]. In addition, there is evidence suggesting a linking between the growth in health inequality and the observed trends in wealth inequality [25].

We found that depressive symptoms are an important aspect when investigating functioning in older adults. It was not only associated with functioning, but also show heterogeneity across household wealth groups among those reporting disability: the odds of the association between disability and depressive symptoms are nearly three times higher among the poorest. Previous results from the Whitehall II Study [5] showed that there is an inverse gradient in both depressive symptoms and in poor functioning by SES and the current findings provide further evidence of the interactive effect of depressive symptoms and SES on functioning. This pathway makes sense, corroborating cohort studies that have explored the effect of depressive symptoms on the onset of disability [18]. On the other hand, since this is a cross-sectional analysis, it is reasonable to consider another pathway: disability [8,9] and lower SES [5,26] leads to depressive symptoms. Previous studies that had explored the association between depressive symptoms and health outcomes [5,11,12,13,15,27,28] reported adjusted coefficients by SES that might represent merely the average coefficients across the SES categories rather than its real meaning considering this interaction. In order to test whether the heterogeneity found was due to a differential intensity of depressive symptoms across SES categories, we performed the Kruskal-Wallis test to see any potential difference between the numbers of positive depressive symptoms by the three disability categories. We found only statistical differences among women, indicating that this apparent interaction might be due to more severe levels of depressive symptoms among the poorest women.

According to psychosocial theory [1,29], disability inequalities linked to depressive symptoms could be partially explained by social support or social integration. Ours findings show that the lack of weekly contact with children, family or a friend and the absence of a partner are related factors among men and, among women, the related factors are loneliness and the absence of a partner. Indeed, it has been noted that those most in need of support from their social networks, such as in the event of disability, are often those least likely to receive support [30]. Our results show that men report lower levels of support from children, family or friends than women. This finding could be explained by the fact that for men social support is heavily focused on their spouses, whereas women are much more likely to rely on a child, close relative, or a friend as their confidant and mobilize more social supports during periods of stress. Therefore, the idea that married people have the best health seems to apply mostly to men and the absence of a partner seems to be more mentally detrimental among men, demonstrated by higher odds of depressive symptoms in all SES groups, regardless of having a lower prevalence amongst women. Social networks, especially partners, could help to attenuate patterns of health inequalities in functioning among older adults [31].

Reviews published recently show that marriage has a protective effect for survival considering younger [32] and older adults [33]. Considering disability, married older adults are less likely to experience ADL decline and more likely to experience ADL recovery [34]. Our results partly corroborate with these findings showing that this occur for both the poorest men and women. Strikingly, for women with disability from the other socioeconomic groups, the pattern is just the opposite: they have more odds to live with a partner compared to the group without disability. As mentioned before, women tend to have more extensive social sources than men, not focusing only on a spouse, as they report more social support from other sources (children, family or friends) in all groups (data not shown). Unmarried women reported their children most frequently as a source of social support in managing a chronic illness [35] and our descriptive analysis show that the wealthiest and poorest groups have a bit more social support from friends, family or children than the disability-free group. These might attenuate the absence of a partner for health-related social control among women. On the other hand, married women tend to accumulate more wealth than single women, which could overinflate the number of married women in more wealthy groups [36].

Old age gives rise to the feeling of loneliness due to the increase in the number of experienced losses. One of these losses, according to ours results, could be disability. We observed that older adults that have reported disability are more likely to report loneliness, independently of SES group. Disability could lead to a feeling of loneliness because difficulties in managing independent daily life impede on engagement in social relationships and fulfillment of social roles and could lead to emotional stress [37]. However, after adjustment for depressive symptoms, reported to be associated with loneliness [10], this association does not apply to all SES groups. This suggest that the link between loneliness and disability/SES status is due to depressive symptoms, despite studies showing that loneliness is itself a risk factor for physical functioning limitations [38]. Poorest women report more loneliness, have more severe depressive symptoms and are more likely to have disability. This pattern is not the same for men. It seems that qualitative psychosocial factors account more for disability among women while quantitative psychosocial factors account more for men.

To the best of our knowledge, this is one of the first studies to explore wealth inequalities in disability taking into account both depression and social support in older adults. The combined effect of psychosocial factors and SES on physical functioning has been explored before, concluding that social participation and living arrangements might alleviate the negative effects of lower SES [39]. Other studies have found that among lower SES groups, psychosocial factors are more significant indicators of self-rated health [30,31] than in higher SES groups. More studies are needed to explore the interaction found between depressive symptoms and SES on disability.

The use of a national sample of community-dwellers and the richness of the data from a well-established study are strengths of our study. The former makes our findings more generalizable to the English population aged 50 years and older, and the latter allowed a better adjustment of confounding and mediating factors. The quality of the measurement of wealth minimised the possibility of measurement bias for SES. Additionally, further adjustment for level of education as another indicator of SES has been done without substantial changes on the results. We are however aware of some limitations. Firstly, due to subjective measures used as exposure and outcome and the measure of SES as outcome, nearly half of participants was excluded of which were older females with disability from the lowest socioeconomic group. This might have generated sub estimated odds ratios for the poorest group and mainly for marginal confidence intervals, such as for loneliness. Secondly, it is also not possible to rule out a same-source bias, that is, the exclusive use of self-reported variables to measure both functioning and social support and loneliness [40]. This bias is difficult to eliminate, since social support and loneliness are by definition subjective evaluations. Finally, it is a cross-sectional design, which impedes establishing temporal relations between the independent variables and the dependent variable.

## Conclusions

Our findings showed a clear wealth gradient in disability with better levels of social resources found among those who were better off. Qualitative psychosocial factors account more for disability among women while quantitative psychosocial factors account more for disability amongst men. The strong inverse dose-response association between wealth and depressive symptoms among participants with disability suggests an interaction, highlighting the importance of prevention and control of depression when making new policies in order to decrease disability and health inequalities. Initiatives to increase social participation and social support among older adults especially those from vulnerable areas and living in care homes should also be encouraged.
